# The Role of Survivin in Podocyte Injury Induced by Puromycin Aminonucleoside

**DOI:** 10.3390/ijms15046657

**Published:** 2014-04-17

**Authors:** Xuejuan Li, Xiaoyan Zhang, Xiaoyan Li, Fangrui Ding, Jie Ding

**Affiliations:** Department of Pediatrics, Peking University First Hospital, No.1 Xi An Men Da Jie, Beijing 100034, China; E-Mails: clairesnow@126.com (X.L.); wserien@163.com (X.Z.); xiaoyanli_520@163.com (X.L.); youngbear@126.com (F.D.)

**Keywords:** survivin, podocyte apoptosis, nephropathy, cyto-protection

## Abstract

**Objective:**

Survivin is a member of the inhibitor of apoptosis protein family, which uniquely promotes mitosis and regulates apoptosis in cancer cells. Recent studies have demonstrated that survivin also expresses in several normal adult cells. In the present study, we aimed to investigate the function of survivin in the terminally differentiated epithelial cells, podocytes.

**Methods:**

Survivin expression and location were detected by Quantitative Real-Time PCR, western blot and fluorescence confocal microscopy methods in normal and injured mouse podocytes. Cyto-protection function of survivin was also studied in cultured podocyte injured by puromycin aminonucleoside (PAN), transfected with survivin siRNA to down-regulate survivin expression, or with survivin plasmid to transiently over-express survivin.

**Results:**

In podocytes, PAN stimulated expressions of survivin and the apoptosis related molecule caspase 3. Knockdown of *survivin* expression by siRNA increased the activation of caspase 3, induced podocyte apoptosis and remarkable rearrangement of actin cytoskeleton. Moreover, over-expression of survivin inhibited PAN-induced podocyte apoptosis and cytoskeleton rearrangement.

**Conclusion:**

Our data provides the evidence that survivin plays an important role in protecting podocytes from apoptosis induced by PAN. The mechanism of survivin related anti-apoptosis may, at least partially, be through the activation of caspase 3.

## 1. Introduction

Survivin (gene name *Birc5*), is a member of inhibitor of apoptosis family, and is the strongest inhibitor of apoptosis factor identified so far [1,2]. The expression of survivin is low or absent in the terminally differentiated normal tissues/cells [3]. Various studies have demonstrated that survivin is over-expressed in cancer tissues/cells [4–9], and the higher expression of survivin plays a significant role in the inhibition of apoptosis [10–13]. The mechanism of apoptosis inhibition by survivin is mainly through directly or indirectly interfering caspase activities in response to apoptosis stimuli [10–14]. The discrepancy of survivin expression in cancers *versus* normal tissues makes survivin as a useful tool for cancer diagnosis and a promising molecular therapeutic target [15,16]. Moreover, several studies report that survivin expresses during embryonic and fetal development and deletion of survivin gene is fatal for embryos [17], suggesting that survivin also has an important function in cell cycle [18,19].

Interestingly, recent studies have demonstrated that survivin expresses in some normal adult cells, including T lymphocytes [20,21], gastric mucosal epithelial cells [22], kidney epithelial cells [23,24] and cardiomyocytes [25,26]. Survivin expression displays a cell specific up-regulation after experimental traumatic brain injury in rats [27]. Survivin over-expression inhibited ethanol-induced gastric epithelial cells apoptosis [28]. In 2013, Terasaki *et al.* also reported that survivin inhibited apoptosis of human lung epithelial cells in acute lung injury, partly by interfering with effector caspases [29]. Disturbing the expression of survivin by anti-sense techniques or generating specific survivin-deficient mice leads to more apoptosis and faster disease progression [30,31].

However, the significance of survivin expression in glomerular podocytes is yet unknown. Podocytes are the highly terminally differentiated epithelial cells critically required for maintenance of glomerular filtration barrier [32]. Several experimental and clinical reports demonstrate that podocyte apoptosis is a key step in the progression of glomerular injury and progression to sclerosis [33–35]. In our previous studies [36], survivin was found to be significantly up-regulated in podocytes of rats with experimental puromycin aminonucleoside (PAN) nephropathy. PAN, a podocyte toxin, is widely used to induce experimental nephrotic syndrome in rats [37–39], and causes foot process effacement and apoptosis in cultured podocytes [40–42]. We hypothesize that survivin has an important role in protecting podocytes from apoptosis induced by PAN. In this study, we investigated the role of survivin in the podocytes injured by PAN.

## 2. Results

### 2.1. Survivin Expression Was Increased in Podocytes after PAN Treatment

**Figure 1.**
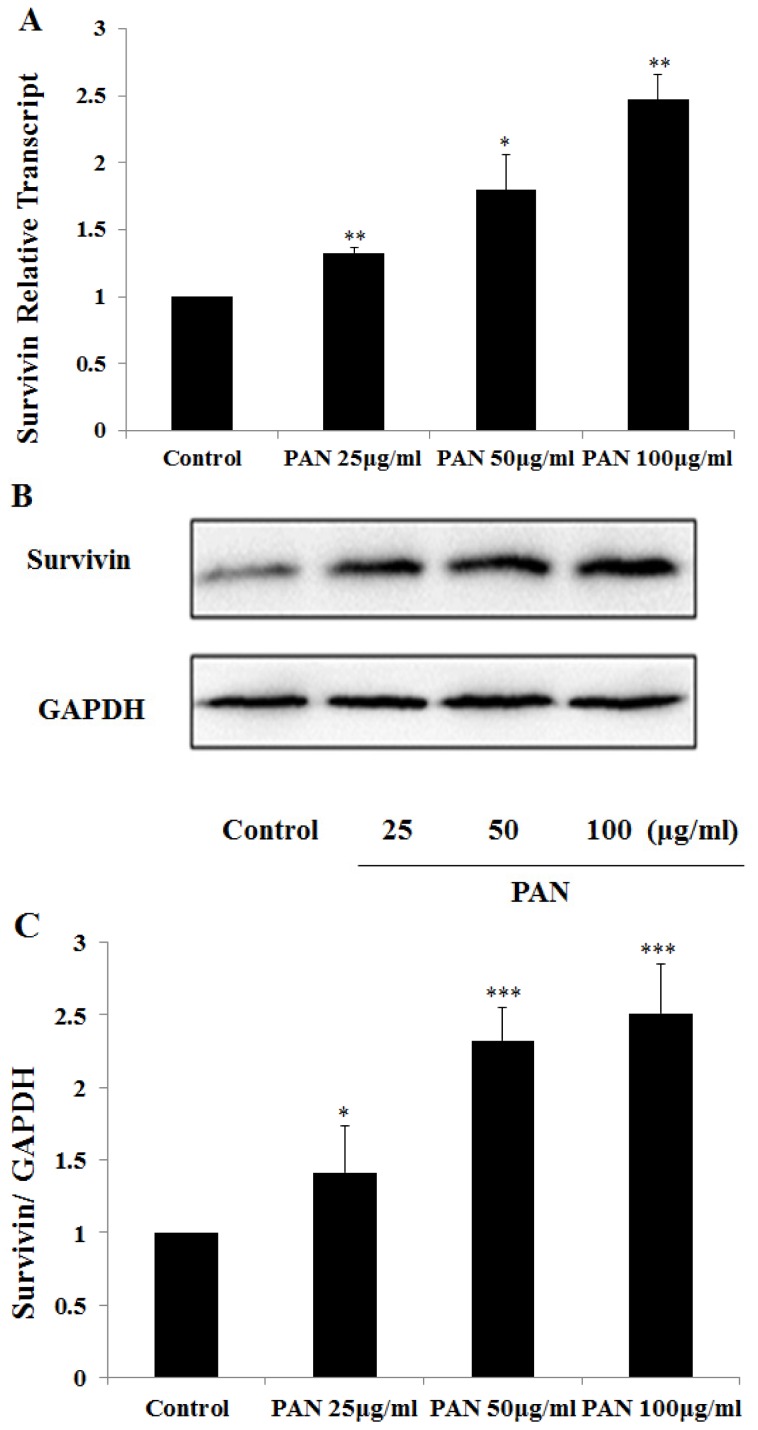
Survivin expression increased in podocytes after puromycin aminonucleoside (PAN) treatment for 24 h. (**A**) The RNA level of survivin was evaluated by the Quantitative Real-Time PCR; (**B**) The protein level of survivin was performed by western blot; (**C**) The amounts of protein were quantified and calibrated with the expression of GAPDH. Data are presented as mean ± SD. *n* = 3. * *p* < 0.05, ** *p* < 0.01, *** *p* < 0.001.

We investigated survivin expression in cultured podocytes treated with puromycin aminonucleoside (PAN). Survivin expression was up-regulated in a dose dependent manner. Survivin mRNA level was increased after normalization by GAPDH (Figure 1A), and survivin protein level reached 1.4, 1.9 and 2.2 folds of the control level in a PAN dose dependent manner (Figure 1B,C). At the same time, in the normal podocytes, survivin fluorescence was weak and evenly distributed in the cytoplasm and nucleus, whereas their fluorescence intensity in PAN treated podocytes increased remarkably in PAN-treated podocytes, further confirming the fact that survivin was up-regulated in the injured podocytes.

### 2.2. PAN Induced Podocyte Apoptosis with Significant Rearrangement of F-Actin

**Figure 2.**
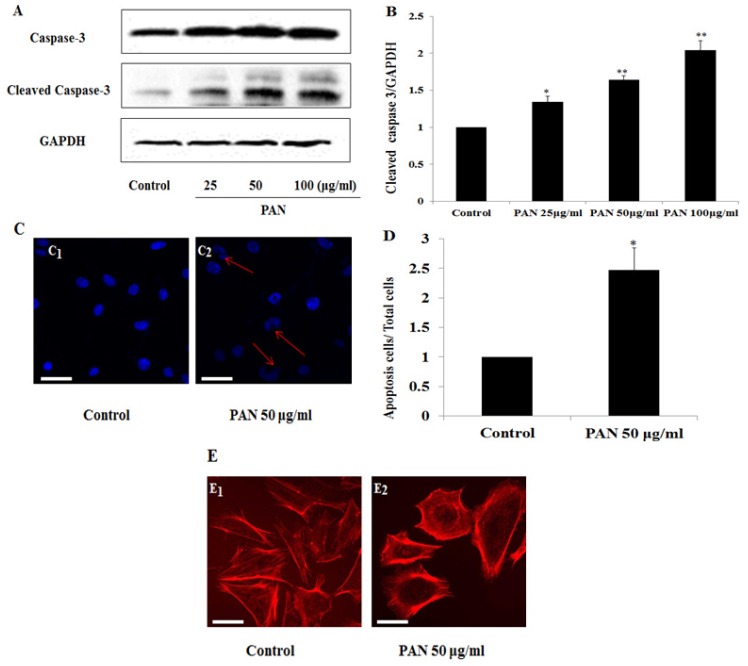
Injury of podocyte after PAN treatment 24 h. (**A**) Cell lysates were analyzed via western blot with antibodies against caspase 3, cleaved caspase 3 and GAPDH, respectively; (**B**) The amounts of protein were quantified and calibrated with the expression of GAPDH; (**C**) Images of podocytes stained with Hoechst (400×, original magnification). Hoechst staining demonstrates nuclei of apoptosis cells and living cells. Red arrows represent apoptotic cells. Bar = 40 μm; (**D**) Quantitative analysis of apoptosis cells presents as apoptosis cells/total cells; (**E**) *F*-actin staining with the fluorescence study (red color). Bar = 40 μm. Data are presented as mean ± SD. *n* = 3. * *p* < 0.05, ** *p* < 0.01.

Caspases are a family of cysteine proteases involving in the crucial processes of apoptosis. We observed the changes of activated caspase 3 in podocytes treated with 25, 50, and 100 μg/mL PAN for 24 h. Activated caspase 3 increased in a PAN dose dependent manner (Figure 2A,B). In addition, we used Hoechst stain analysis to detect nuclear changes in apoptotic cells, which were significantly increased in podocytes after treatment with PAN 50 μg/mL for 24 h (Figure 2C,D). The distribution of *F*-actin had an obvious rearrangement in those cells by fluorescence confocal microscopy (Figure 2E). We then used 50 μg/mL PAN as the following experiments.

### 2.3. Knockdown of Survivin Expression Exacerbated PAN-Induced Injury of Podocytes

**Figure 3.**
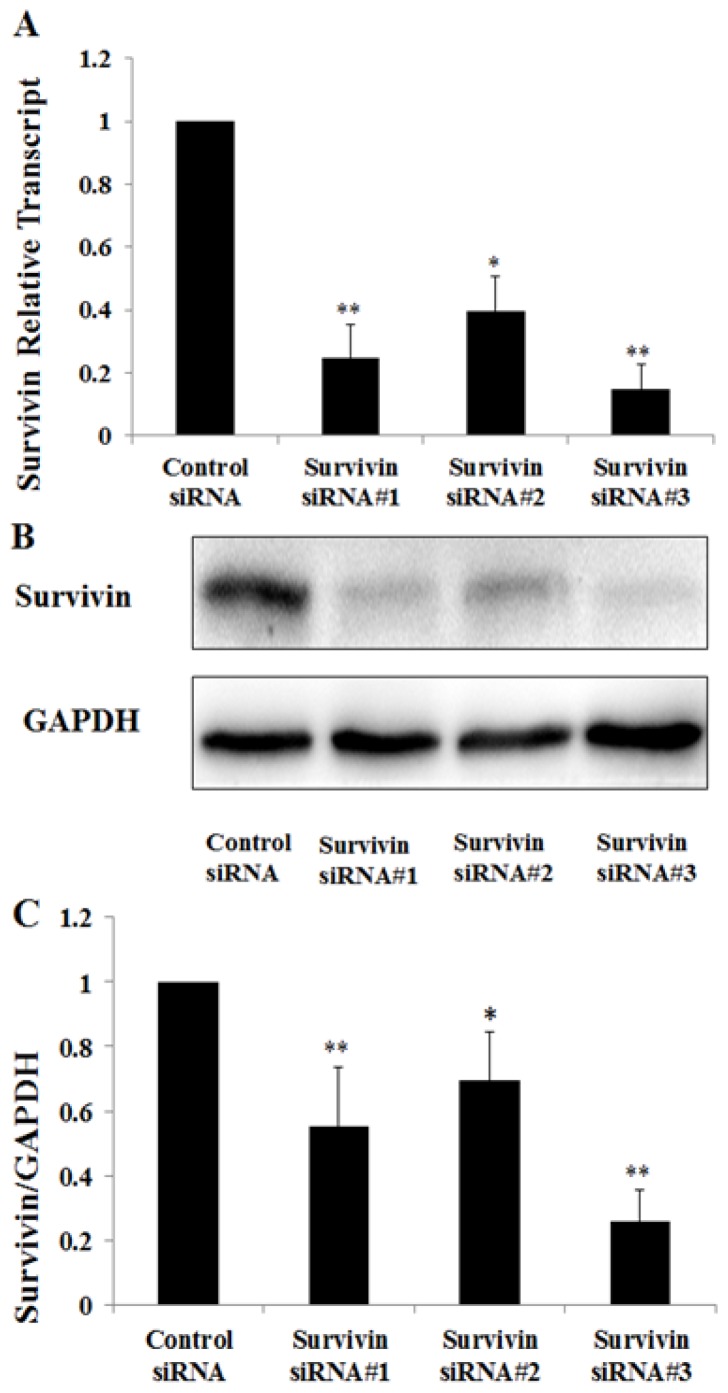
Screening of high effective survivin siRNA. The total RNA (**A**) and protein (**B**,**C**) expression of survivin decreased significantly after transfection with survivin siRNA 100 pmols compared to negative control siRNA, especially survivin siRNA#3 group. Data are presented as mean ± SD. *n* = 3. * *p* < 0.05, ** *p* < 0.01.

**Figure 4.**
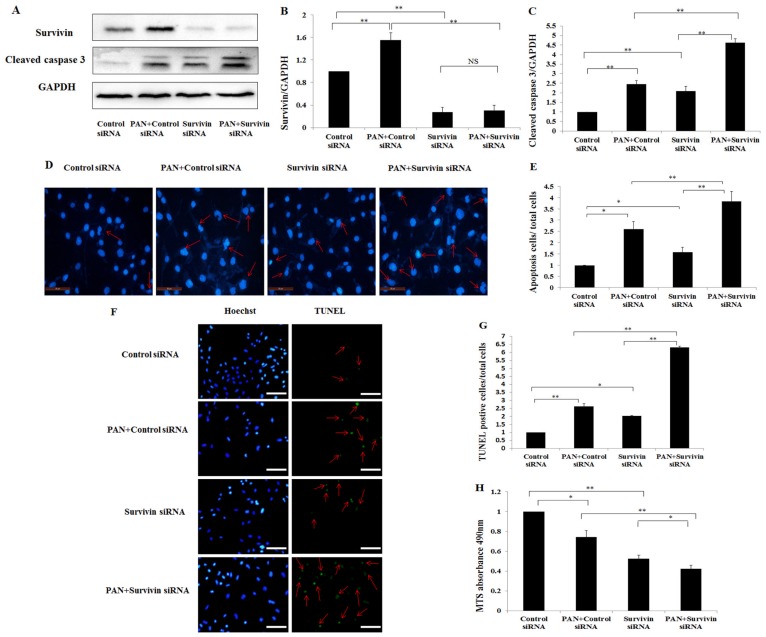

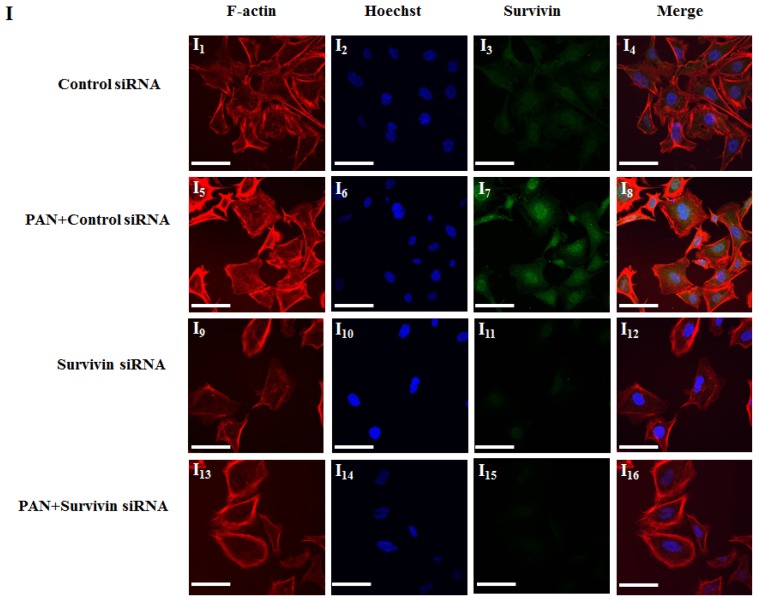
The effect of knocked down survivin on PAN-induced injury of poodcytes. (**A**) Total cell lysates were prepared and analyzed via western blot with antibodies against survivin, cleaved caspase 3 and GAPDH respectively; (**B**,**C**) The amounts of each protein were quantified and calibrated with the expression of GAPDH; (**D**) Images of podocytes stained with Hoechst (200×, original magnification). Hoechst staining shows nuclei of apoptosis and living cells, Bar = 50 μm. Red arrows indicate apoptotic cells; (**E**) Quantitative analysis of apoptosis cells presents as apoptosis cells/total cells; (**F**) Images of podocytes stained with TUNEL. Bar = 100 μm. The green color is indicative of TUNEL-positive cells (indicated with red arrows), and the blue color marks the presence of all cells; (**G**) The percentage of apoptotic cells is reported. For each group in a given experiment, at least 300 randomly chosen cells were analyzed; (**H**) MTS analysis of the rate of podocytes viability; (**I**) *F*-actin and survivin staining with the fluorescence study demonstrates in red and green, respectively. The double-labelled assays showed that survivin co-localized with *F*-actin. Bar = 40 μm. Data are presented as mean ± SD. *n* = 3. * *p* < 0.05, ** *p* < 0.01, NS means no significance.

To explore the role of survivin in PAN induced podocyte injury, we down-regulated survivin expression in podocytes by using siRNA and then analyzed caspase 3 expression in these cells by western blot. The down-regulation of survivin mRNA by siRNA was examined by Quantitative Real-Time PCR and western blot. Survivin siRNA #1, #2, and #3 down-regulated survivin mRNA by 55%, 28% and 75%, respectively (Figure 3A), and decreased survivin protein by 75%, 61% and 85%, respectively (Figure 3B,C). Therefore, survivin siRNA 3# was used for the following experiments. Up-regulation and activation of caspase 3 were found in podocytes treated with survivin siRNA, and were significantly enhanced in podocytes treated with PAN + survivin siRNA (Figure 4A,C). Consistent with the increase of activated caspase 3 after down-regulation of survivin, Hoechst staining of cell nuclei (Figure 4D,E) and TUNEL assay (Figure 4F,G) also demonstrated a significant increase of apoptotic cells number. To further observe the cell viability in down-regulation of survivin, we detected the rate of podocyte viability by MTS and the viability was significantly decreased (Figure 4H). In addition, the integrity of the cytoskeleton is important to maintain the function and morphology of podocytes. Rearrangement of F-actin was found in those cells by fluorescence confocal microscopy. In podocytes transfected with negative control siRNA, the *F*-actin was distributed as stress fiber-like bundles along the axis of podocytes (Figure 4I_1_). Survivin knockdown podocyte showed rearrangement of *F*-actin (Figure 4I_9_), which was more severe in podocytes treated with PAN + survivin siRNA (Figure 4I_13_).

### 2.4. Over-Expression of Survivin Expression Ameliorated PAN-Induced Injury of Podocyte

**Figure 5.**
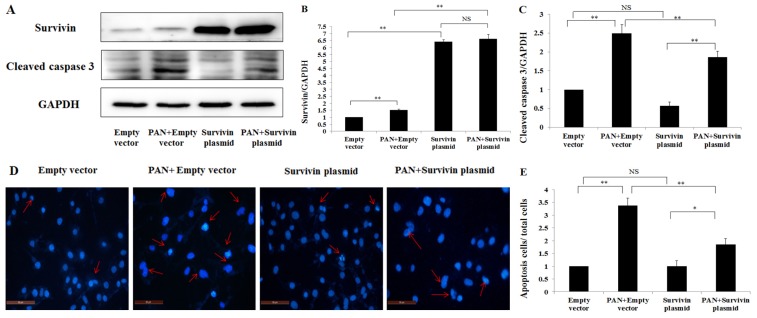

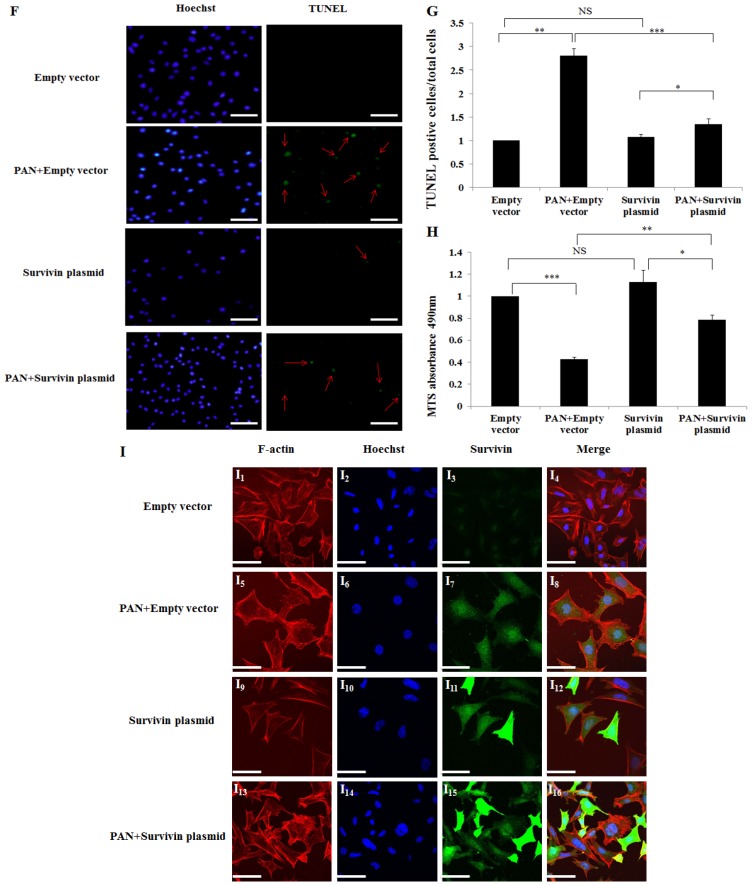
The effect of over-expressed survivin on PAN-induced injury of poodcytes. (**A**) Total cell lysates were analyzed via western blot with antibodies against survivin, cleaved caspase 3 and GAPDH; (**B**,**C**) The amounts of each protein were quantified and calibrated with the expression of GAPDH; (**D**) Images of podocytes stained with Hoechst (200×, original magnification), Bar = 50 μm. Hoechst staining shows nuclei of apoptosis and living cells. Red arrows indicate apoptotic cells; (**E**) Quantitative analysis of apoptosis cells was shown as apoptosis cells/total cells; (**F**) Images of podocytes stained by TUNEL. Bar = 100 μm. The green color is indicative of TUNEL-positive cells (indicated with red arrows), and the blue color marks the presence of all cells; (**G**) The percentage of apoptotic cells is reported. For each group in a given experiment, at least 300 randomly chosen cells were analyzed; (**H**) MTS analysis of the rate of podocytes viability; (**I**) *F*-actin and survivin staining with the fluorescence study demonstrates in red and green, respectively. The double-labelled assays showed that survivin co-localized with *F*-actin. Bar = 40 μm. Data are presented as mean ± SD. *n* = 3. * *p* < 0.05, ** *p* < 0.01, *** *p* < 0.001, NS means no significance.

To further explore the protective effect of survivin in PAN-induced injury of podocytes, podocytes were transfected with pCMV6-Kan/Neo-survivin plasmids (survivin plasmid) to over-express survivin. The over-expression efficiency was analyzed by western blot (Figure 5A,B). Survivin over-expression by transfection of survivin plasmid induced the decrease of activated caspase 3 in podocytes and also in podocyte treated with PAN (Figure 5A,C). Consistent with the decrease of activated of caspase 3 after survivin over-expression, Hoechst stain assay (Figure 5D,E) and TUNEL assay (Figure 5F,G) also demonstrated a significantly lower number of apoptotic cells compared with PAN + empty vector group. To further confirm the reduced apoptosis in survivin over-expression group, we detected the rate of podocytes viability by MTS assay, and the viability was significantly increased in the podocyte over-expression group comparison with the Empty vector group injured by PAN (Figure 5H). In addition, the *F*-actin had an obvious recovered arrangement by fluorescence confocal microscopy. In the empty vector group, *F*-actin was characterized by the presence of highly ordered parallel, contractile actin filament bundles (Figure 5I_1_). *F*-Actin was present as disordered manner, showing reorganized, short, branched actin filaments filled in cytoplasm in podocytes treated with PAN (Figure 5I_5_), while PAN + survivin plasmid group partly recovered the normal arrangement *F*-actin (Figure 5I_13_).

## 3. Discussion

In this study, survivin expression was up-regulated both in mRNA and protein levels in a dose dependent manner induced by PAN. The increase of survivin was also reported in our previous study. In our previous study, survivin expression was significantly increased, both in rat PAN nephropathy and in patients with proteinuric renal diseases including minimal change nephrotic syndrome (MCNS), focal segmental glomerulosclerosis (FSGS) and membranous nephropathy (MN) [36]. All of these results indicated that survivin was involved in the processes of podocyte injury. Podocyte are the key target cells of injury in a variety of renal diseases, especially proteinuric renal diseases, such as MCNS, FSGS and MN, *etc*. Podocyte apoptosis is one of the major and important phenomena of podocyte injury. The expression of survivin in podocyte was also observed in a previous study [24], in which survivin expression was detected in normal kidney tissues of adult rats. Cells expressing survivin co-localized with synaptopodin in consecutive sections, which implies that survivin expresses in podocytes. However, the significance of increased expression of survivin in injured podocyte and the role of survivin in podocyte apoptosis were unknown.

Survivin is a member of the inhibitor of apoptosis protein (IAP) family. A series of studies have identified that survivin is the strongest inhibitor of apoptosis [1,2]. In the present study, we detected that survivin expression was increased in injured podocytes. In addition, Hoechst stain demonstrated the increase of apoptosis in PAN treated podocyte (Figure 2C,D). Podocyte apoptosis was also detected and reported by several other studies [43–47]. In present study, significant apoptosis was found in podocytes treated with PAN and survivin siRNA (Figure 4C,E,G). However, several studies demonstrated that higher level of apoptosis was detected in cancer cells after drug treatment [48]. The possible reason is that podocytes are relatively not sensitive to drug treatment compared to cancer cells. We performed knockdown and over-expression of *survivin* in normal and PAN injured podocytes to disclose the role of survivin in the processes of podocyte apoptosis. Knockdown *survivin* rendered podocytes susceptible to PAN induced injury, increased the number of apoptotic cells (Figure 4D–G), decreased the viability of podocytes (Figure 4H), and induced obvious rearrangement of *F*-actin. Over-expression of survivin by transfection survivin plasmid ameliorated PAN induced podocytes injury, showing lower numbers of apoptotic cells (figure 5D–G), better viability of podocytes (Figure 5H), and nearly normal arrangement of *F*-actin. These results indicate that knockdown the expression of *survivin* in podocytes leads to greater susceptible to injury factors. Several other studies also found the similar results in other cell types. For example, knockdown *survivin* with small interfering RNA rendered human lung epithelial cells susceptible to bleomycin-induced cell damage [29], and led to a significant decrease in the number of viable ectopic ESCs following staurosporine treatment in human endometriotic stromal cells [49]. Specific deletion of survivin markedly delayed recovery of the kidney ischemia-reperfusion (I/R) injury in mouse renal proximal tubule cells [50]. On the other hand, the over-expression survivin in podocytes increased the resistance to PAN induced injury. In 2008, over-expression of survivin in cardiomyocytes by adenovirus mediated method inhibited doxorubicin-induced apoptosis [31]. And in 2013, Terasaki *et al.* demonstrated that over-expression of survivin decreased bleomycin-induced damage in human lung epithelial cells [29]. Our results suggested that survivin could protect podocytes from apoptosis induced by PAN, and survivin in podocytes may play an anti-apoptosis role.

The anti-apoptosis mechanism of survivin has not been completely understood [51]. Our results showed that down-regulation of survivin resulted in the increase of activated of caspase 3, and over-expression of survivin had an anti-apoptotic role by inhibiting the activation of caspases 3 (Figures 4A,C and 5A,C). Thus, caspases may be involved in the mechanism of survivin related anti-apoptosis. Several studies by other groups suggested that survivin inhibited cell apoptosis mainly through interfering with caspase dependent manner [10–12]. Survivin may specifically bind to the terminal effector cell death proteases, caspase 3 and 7 and inhibited caspase activity in cancer cells [10]. In recent years, studies demonstrated that survivin may also play a role in inhibiting the caspase-independent apoptosis in cancer cells [52,53], and that down-regulated survivin induced the translocation of apoptosis inducing factor (AIF) from the cytoplasm to the nucleus in cancer cells, while caspase 3 activity showed no change.

In the present study we have detected the increase survivin expression in podocytes after PAN treatment but the role of survivin in podocytes is not completely understood. However, our study indeed demonstrates for the first time that down-regulation of survivin expression exacerbated podocyte apoptosis and the over-expression of survivin ameliorated the PAN induced apoptosis.

## 4. Experimental Section

### 4.1. Podocyte Culture

Immortalized mouse podocytes (MPC5, gift from Peter Mundel, Boston, MA, USA) were cultured under growth-permissive conditions on rat tail collagen type I-coated plastic dishes (BD Bioscience, Franklin Lakes, NJ, USA), at 33 °C in RPMI 1640 medium (Invitrogen, Carlsbad, CA, USA) supplemented with 10% fetal bovine serum (Gibco BRL, Gaithersburg, MD, USA), 10 U/mL mouse recombinant γ-interferon (Sigma, St. Louis, MO, USA), and 100 U/mL penicillin plus 0.1 mg/mL streptomycin (Gibco BRL, Gaithersburg, MD, USA). To induce differentiation, podocytes were maintained in non-permissive conditions at 37 °C without γ-interferon for 14 days, and used for the experiments. PAN is widely used to study renal diseases by inducing nephrotic syndrome *in vivo* and podocyte injury *in vitro* [54,55]. Therefore, different concentrations of PAN (25, 50, 100 μg/mL, Sigma) were used to cause podocyte injury. Both mRNA and protein were collected after 24 h stimulation. All experiments were performed in triplicates.

### 4.2. Quantitative Real-Time PCR

Total RNA was isolated from cultured podocytes by using the Trizol reagent (Invitrogen). Two micrograms of RNA were reversely transcribed using the high capacity cDNA Reverse Transriptase kit (Invitrogen) following the manufacture’s protocol. Primers used in Quantitative Real-Time PCR included: 5′-CGGAGTCAACGGATTTGGTCGTAT-3′ (sense) and 5′-AGCCTTCTCCATGGTGGT GAAGAC-3′ (antisense) for GAPDH cDNA, and 5′-ATCGCCACCTTCAAGAACTG-3′ (sense) and 5′-CAGGGGAGTGCTTTCTATGC-3′ (antisense) for survivin cDNA. Real-time PCR amplification was performed using the SYBR Green PCR Master Mix Kit (Invitrogen, Carlsbad, CA, USA). Cycling conditions included denature at 95 °C for 10 min followed by annealing at 40 repeats of 95 °C for 15 s and extension at 58 °C for 1 min. Relative quantity of mRNA were normalized by GAPDH and calculated using the delta-delta method from threshold cycle numbers. On the basis of exponential amplification of target gene as well as calibrator, the amount of amplified molecules at the threshold cycle is given by 2^−ΔΔ^*^C^*^t^.

### 4.3. Western Blot

Podocytes were lysed with a RIPA buffer containing protein inhibitors (1 mM phenylmethylsulfonyl fluoride, 1 μg/mL leupeptin and pepstatin). Thirty micrograms of the total protein were subjected to 8%–15% SDS-PAGE and transferred to nitrocellulose membranes (Amersham biosciences, Piscataway, NJ, USA). After blocking with PBS containing 5% nonfat dry milk for 1 h at room temperature, then incubated overnight at 4 °C with the following primary antibodies, rabbit anti-survivin (1:2000, Abcam, Cambridge, MA, USA), rabbit Anti-cleaved caspase 3 (1:750, Cell Signaling Technology, Beverly, MA, USA) and mouse Anti-GAPDH (1:5000, Chemicon, Temecula, CA, USA) antibody at 4 °C over night (14–16 h). Subsequently, the membranes were rinsed three times, each time for 10 min in PBS buffer with 0.05% Tween-20 and incubated with horseradish peroxidase-conjugated anti-rabbit or mouse IgG (Santa Cruz Biotechnology, Santa Cruz, CA, USA). After a final washing, the membranes were developed using an enhanced chemiluminescence reagent (Millipore, Bedford, MA, USA), and the specific protein bands were scanned and quantitated in relation to GAPDH. The densitometric analysis of images was performed using Image J software (National Institute of Mental Health, Bethesda, MD, USA).

### 4.4. Small Interfering RNA (siRNA) Experiment

Synthetic siRNA targeting mouse survivin and non-targeting control siRNA were obtained from RiboBio. The target sequences of double-stranded nucleotides used for siRNA knockdown are 5′-CGATAGAGGAGCATAGAAA-3′ for survivin (#1), 5′-CCGAGAACGAGCCTGATTT-3′ for survivin (2#), 5′-CCGTCAGTGAATTCTTGAA-3′ for survivin (#3) (RIBOBIO, Guanzhou, China). Transfection was performed with Lipofectamine RNAi MAX reagent (Invitrogen), according to the manufacturer’s protocol. Forty-eight hours after transfection, cells were treated with PAN for 24 h. The total protein extracts from the cells were used for western blot analysis.

### 4.5. Survivin Over-Expression Experiment

The plasmid-encoding mouse survivin pCMV6-Kan/Neo-survivin and the empty vector pCMV6-Kan/Neo were purchase from Origene (Origene, Rockville, MD, USA). Podocytes were transiently transfected with a survivin expression plasmid pCMV6-Kan/Neo-survivin and the empty vector pCMV6-Kan/Neo using lipofectamine 2000 Transfection Reagent (Invitrogen) according to the manufacturer’s instructions. Meanwhile, 48 h after transfection, cells were exposed to PAN for 24 h. Transfection efficiency was controlled by western blot.

### 4.6. Cell Viability Assay

The cell viability was measured by MTS reduction activity. Briefly, cells transfected with siRNA and the negative control siRNA or survivin plasmid and empty vector were seeded in a 96-well plate, incubated with 50 μg/mL PAN for 24 h, and then with 38 μg/mL MTS (Promega, Madison, WI, USA) for 3 h at 37 °C. The absorbance at 490 nm was read using a microplate reader (BioTek, Winooski, VT, USA).

### 4.7. Hoechst 33258 Staining

After treatment, cells were washed three times with phosphate buffered saline (PBS) and stained with a DNA specific dye, Hoechst 33258 (Sigma). The cells were viewed under a fluorescence microscope (Olympus, Tokyo, Japan). Characteristic apoptotic morphology such as, chromatin condensation and DNA fragmentation was observed after Hoechst staining, while nuclei of non-apoptotic cells stained homogenous blue color due to the evenly spread and mono-granulated chromatin.

### 4.8. Terminal Deoxynucleotidyl Transferase dUTP Nick-End Labeling (TUNEL)

The TUNEL assay was performed using a commercial fluorometric TUNEL system kit (Promega) according to the manufacturer’s instructions. Podocytes were plated at a density of 1.5 × 104 cells per dish with coverslips bottoms. Cells were transfected with siRNA and negative control siRNA or survivin plasmid and empty vector, and then treated with 50 μg/mL PAN for 24 h. Nuclear with Hoechst staining (Sigma) and TUNEL staining was examined under fluorescence microscope (Olympus). For each group in a given experiment, at least 300 randomly chosen cells were analyzed.

### 4.9. Fluorescence Confocal Microscopy

On the coverslip’s podocytes were fixed with 4% paraformaldehyde, followed by permeabilization and blocking with 0.3% Triton X-100 and 10% goat serum. Rabbit anti-survivin (1:200, Abcam) was used as the primary antibody. For *F*-actin staining, fixed and permeabilized cells were incubated with Alexa-phalloidin (1:200, Invitrogen). After three washes with PBS, the slides were incubated with Alexa Fluor^®^ 488 Goat Anti-Rabbit IgG (1:200, Invitrogen). Hoechst nuclear dye was applied. The slides were mounted with 15% Mowiol (Sigma). Stained images for each antibody at the same light exposure were obtained by confocal laser-scanning microscopy (Zeiss Lsm510 Meta, Jena, Germany). Photographs of podocytes stained with each antibody were selected randomly and analyzed by a person who was blinded to the study groups.

### 4.10. Statistical Analysis

The statistically significant difference among means of four groups was determined by one-way analysis of variance. An unpaired, two-tailed Student’s *t*-test was used to determine significant differences between the two groups, *p* < 0.05.

## 5. Conclusions

Our data provides evidence that survivin plays an important role in protecting podocytes from apoptosis in PAN-induced injury. The potential anti-apoptosis mechanism of survivin may relate to caspase 3. Survivin may be an essential mediator of cyto-protection in podocytes injury. Furthermore, whether survivin could be a potentially molecular target for treating proteinuric diseases still needs investigation.
